# Oxidative Stress in Wild Boars Naturally and Experimentally Infected with *Mycobacterium bovis*

**DOI:** 10.1371/journal.pone.0163971

**Published:** 2016-09-28

**Authors:** Diana Gassó, Joaquín Vicente, Gregorio Mentaberre, Ramón Soriguer, Rocío Jiménez Rodríguez, Nora Navarro-González, Asta Tvarijonaviciute, Santiago Lavín, Pedro Fernández-Llario, Joaquim Segalés, Emmanuel Serrano

**Affiliations:** 1 Servei d´Ecopatologia de Fauna Salvatge (SEFaS), Departament de Medicina i Cirurgia Animals, Universitat Autònoma de Barcelona (UAB), Bellaterra, Spain; 2 Sabio-IREC Instituto de Investigación en Recursos Cinegéticos (CSIC-UCLM-JCCM), Ciudad Real, Spain; 3 Estación Biológica de Doñana, Consejo Superior de Investigaciones Científicas (CSIC), Sevilla, Spain; 4 CIBER Epidemiología y Salud Pública (CIBERESP), Spain; 5 Western Center for Food Safety, University of California Davis, Davis, California, United States of America; 6 Departament de Medicina i Cirurgia Animals, Universitat Autònoma de Barcelona (UAB), Bellaterra, Spain; 7 Innovación en Gestión y Conservación de Ungulados S.L., Cáceres, Spain; 8 UAB, Centre de Recerca en Sanitat Animal (CReSA, IRTA-UAB), Campus de la Universitat Autònoma de Barcelona, 08193 Bellaterra, Spain; 9 Departament de Sanitat i d’Anatomia Animals, Universitat Autònoma de Barcelona, Bellaterra, Spain; 10 Centre for Environmental and Marine Studies (CESAM), Departamento de Biología, Universidade de Aveiro, Aveiro, Portugal; Institut national de la santé et de la recherche médicale - Institut Cochin, FRANCE

## Abstract

Reactive oxygen and nitrogen species (ROS-RNS) are important defence substances involved in the immune response against pathogens. An excessive increase in ROS-RNS, however, can damage the organism causing oxidative stress (OS). The organism is able to neutralise OS by the production of antioxidant enzymes (AE); hence, tissue damage is the result of an imbalance between oxidant and antioxidant status. Though some work has been carried out in humans, there is a lack of information about the oxidant/antioxidant status in the presence of tuberculosis (TB) in wild reservoirs. In the Mediterranean Basin, wild boar (*Sus scrofa*) is the main reservoir of TB. Wild boar showing severe TB have an increased risk to *Mycobacterium* spp. shedding, leading to pathogen spreading and persistence. If OS is greater in these individuals, oxidant/antioxidant balance in TB-affected boars could be used as a biomarker of disease severity. The present work had a two-fold objective: i) to study the effects of bovine TB on different OS biomarkers (namely superoxide dismutase (SOD), catalasa (CAT), glutathione peroxidase (GPX), glutathione reductase (GR) and thiobarbituric acid reactive substances (TBARS)) in wild boar experimentally challenged with *Mycobacterium bovis*, and ii) to explore the role of body weight, sex, population and season in explaining the observed variability of OS indicators in two populations of free-ranging wild boar where TB is common. For the first objective, a partial least squares regression (PLSR) approach was used whereas, recursive partitioning with regression tree models (RTM) were applied for the second. A negative relationship between antioxidant enzymes and bovine TB (the more severe lesions, the lower the concentration of antioxidant biomarkers) was observed in experimentally infected animals. The final PLSR model retained the GPX, SOD and GR biomarkers and showed that 17.6% of the observed variability of antioxidant capacity was significantly correlated with the PLSR X’s component represented by both disease status and the age of boars. In the samples from free-ranging wild boar, however, the environmental factors were more relevant to the observed variability of the OS biomarkers than the TB itself. For each OS biomarker, each RTM was defined as a maximum by one node due to the population effect. Along the same lines, the *ad hoc* tree regression on boars from the population with a higher prevalence of severe TB confirmed that disease status was not the main factor explaining the observed variability in OS biomarkers. It was concluded that oxidative damage caused by TB is significant, but can only be detected in the absence of environmental variation in wild boar.

## Introduction

Oxidative stress (OS) results from a disturbance of the balance between the production of reactive oxygen (ROS) or nitrogen (RNS) species and the organism's ability to compensate for their damaging effects [1]. During an infectious process, macrophages and neutrophils produce large amounts of ROS and RNS for pathogen clearance [2]. However, these biochemical products do not discriminate between pathogens and the host's own biological structures causing cell injury, triggering physiologic disorders and promoting the pathological process [3]. Since the OS response integrates both the activation of immune response and the ability of the organism to compensate for infection damage [4], it is widely used as a biomarker for assessing the physiological cost of infection in human [1] and animal health [5, 6].

A wide variety of studies have assessed oxidative damage in a broad range of pathologies such as tuberculosis (TB). Immunocompetent hosts infected with *Mycobacterium* spp. mount an immune response mainly based on T cell activation. Although a strong immune response is usually sufficient to control TB, infected hosts rarely clear the bacterium [7]. One reason for its long persistence in an immunocompetent host is that *Mycobacterium* spp. is able to persist within macrophages through diverse evasion strategies, including the inhibition of macrophage RNS production [8]. In a murine model, these inhibitory effects result in the reactivation of persistent *Mycobacterium* spp. infection [9]. On the other hand, TB-affected individuals with poor antioxidant defences suffer from tissue damage due to OS [10, 11]. In fact, inflammation caused by OS in TB-affected patients has been implicated in the pathogenesis of lung fibrosis and dysfunction [12]. In view of this, it seems clear that a proper oxidant/antioxidant balance is essential for the containment of both acute and chronic TB in mammals.

To date, no data have been reported regarding oxidant/antioxidant status in wild reservoirs of the *Mycobacterium tuberculosis* complex (MTC). In the Mediterranean Basin, wild boar (*Sus scrofa*) is the main reservoir of TB [13], one of the major diseases of domestic animals throughout the world. This wild pig can suffer from a severe form of the disease showing macroscopic lesions in cervical lymph nodes, lungs, liver, kidneys or testicles [14]. As described for other host models [15], wild boar suffering from severe TB are considered super-shedders [16]. Wild boar live at different population densities in a wide range of environmental conditions, which may affect the values of OS biomarkers. However, only preliminary work has explored OS patterns in this mammal [17]. Consequently, the wild boar is an excellent model for exploring how OS is affected by environmental and population sources of variation aside from disease occurrence. Furthermore, OS is interesting from an ecological perspective because of the link between OS and the fitness components of organisms [18, 19]. In fact, there is clear evidence about the negative effects of oxidative stress on the reproduction [20], and survival rates [21] of wild vertebrates. For that reason, OS biomarkers can be excellent indicators not only to assess health status [22] but also to measure the impact of environmental variation on individuals and populations.

In spite of the plethora of methods used to determine oxidative damage and antioxidant defences, little information exists about the reliability of these biomarkers to assess the impact of infectious diseases on free-ranging vertebrates. Moreover, the interpretation of OS biomarkers is not a trivial issue. In fact, the proper assessment of oxidative status must take into account not only the concentration of antioxidant defences (i.e., antioxidant vitamins and other substances, and/or up-regulation of endogenous antioxidants (enzymes)), but also oxidative damage [22]. For instance, an elevation of endogenous or exogenous antioxidants with stable oxidative damage concentrations suggests that individuals are successfully dealing with the new oxidative conditions. In contrast, the depletion of antioxidant substances and stable oxidative conditions suggest the physiological exhaustion to cope with the stressor and the risk of oxidative damage.

In addition, the pure effects of a given stressor on the OS biomarkers are hardly estimated because of both individual (e.g., growth [23], genetic diversity [24], reproductive status [25] or immune response [26, 27]), and environmental variation [26]. To complicate matters, all of the previously mentioned factors can act in synergy [28], making the comparison of OS biomarkers between populations and seasons [29] difficult. Surprisingly, there is little information about these limitations in the use of OS biomarkers for most vertebrate species.

In the present work, we studied oxidative status and oxidative defences in 120 wild boar (*Sus scrofa*) living under contrasting environmental conditions. They ranged from dewormed, supplementally fed animals experimentally challenged with *Mycobacterium bovis* to free-ranging individuals inhabiting areas affected by the disease. According to recent recommendations [22] a broad panel of biomarkers of antioxidant and oxidative status were used. We assessed the concentration of four endogenous antioxidant enzymes (AE): superoxide dismutase (SOD), catalase (CAT), glutathione peroxidase (GPX) and glutathione reductase (GR). We also measured concentrations of a biomarker of lipid peroxidation (thiobarbituric acid reactive substances, TBARS). The study had a twofold objective: i) to assess the effect of *M*. *bovis* infection on the oxidant/antioxidant status of wild boar showing different degrees of disease severity, and ii) to evaluate the importance of individual (body weight and gender) and environmental (population and season) sources of OS variation when assessing oxidant/antioxidant status in free-ranging wild boar populations affected by TB.

Since TB is a chronic infection causing a host inflammatory response [30], which in turn has been linked to both OS and pathogenesis of lung fibrosis in human [12, 10] and animal models [11], we expected to find higher OS values in TB-infected wild boar, in particular in those individuals showing gross lesions. On the other hand, because of the effect of external sources of OS, we expected that the relationship between TB and OS would be more difficult to detect in free-ranging animals.

## Material and Methods

### Study areas

Two different populations of free-ranging wild boars were studied, originating in Eastern (The National Game Reserve Ports de Tortosa i Beseit, NGRPTB) and Central (Ciudad Real, CR) Spain. The NGRPTB (40°48’N, 0°19’E; about 28% of the surface area is higher than 1000 m.a.s.l, with the highest peak being Mont Caro (1442 m) and the lower altitudes around 300 m.a.s.l.) is a rough limestone mountain massif of 28,587 ha, with a typical Mediterranean climate and annual mean temperatures (year 2014) of 10.6°C (min = 0.0°C in February, max = 23.4°C in July) and a mean yearly cumulative rainfall (period 2010–2014) of 1084.4 mm (min = 0.1 mm in Setember-August, max = 512.5 mm in November, information kindly provided by *Servei Meteorològic de Catalunya*, www.meteocat.com). CR is located in central Spain (38°55’N, 0°36’E; 600–850 m.a.s.l.) and shows a continental Mediterranean climate with annual mean temperatures (year 2014) of 17.92°C (min = -3.4°C in December, max = 40.6°C in July) and a mean yearly cumulative rainfall (period 2009–2014) of 468.2 mm (min = 0 mm in July, max = 180.2 mm in March, information provided by www.meteociudadreal.com).

### Sampling of free-ranging wild boar

Sixty-one hunter-harvested wild boar from 3 to 80 months of age were sampled in the NGRPTB (n = 24) and CR (n = 37), during the regular hunting season. After animal collapse, the gender of the boar was assigned visually by inspecting genitalia, and age was assessed by recording dental eruption patterns [31]. Additionally, body weight (with viscera) of animals was measured with a dynamometer (precision of 0.1 kg). A necropsy examination of animals was performed to assess the presence of TB-like gross lesions affecting lymph nodes (submandibular, retropharyngeal, mediastinal and mesenteric lymph nodes), and thoracic or abdominal organs [14]. Submandibular and/or retropharyngeal lymph nodes and blood (in separation serum tubes) were collected and stored at 4°C until processing within the following 24 hours.

### Ethics statement

For hunter-harvested boars, no approval was needed from an Ethics Committee since the animals were not culled for research purposes. These animals were legally hunted in their own habitat by authorised gamekeepers and hunters within the framework of an annual hunting plan approved by the local environmental agencies: *Departament d’Agricultura*, *Ramaderia*, *Pesca*, *Alimentació i Medi Natural—Generalitat de Catalunya* (for NGRPTB), and the *Departamento de Medio Ambiente de Castilla la Mancha* (for CR). On the other hand, the approval for culling wild boars used for the experimental *M*. *bovis* infection was given by the Committee on Ethics of Animal Experiments of the Regional Agriculture Authority of CR, Permit number 2741–2009.

### Experimental Mycobacterium bovis infection design

Wild boar (n = 59, 42 males and 17 females, ranging from six to 24 months of age) were purchased from a local TB-free commercial farm at 3–4 months of age and were used in experimental *M*. *bovis* infection trials (previously published in [32] and [33]). Farmed animals were negative for both *Mycobacterium* spp. by ELISA tests [34], regularly performed by veterinary authorities, and to mycobacterial lesions at slaughter. Moreover, farmed wild boar were dewormed yearly and reared under regular veterinary inspection.

The animals were housed in biosecurity level 3 bio-containment facilities where they had *ad libitum* food and water (during three experimental periods October 2010, January 2011 and March 2011; for more information see [33]), and 44 of them were infected with 5 ml of a suspension containing 10^6^ colony forming units (CFU) of a *M*. *bovis* field strain (SBO339 described in [32]). Non- inoculated boars were used as control animals (n = 15). The animals were handed four times during the experiment, where blood samples were collected (days 11, 46, 186 and 300 dpi). Only serums of the last day were used for this propose, the other samples were used for other studies. At the end of the experiment, animals were anaesthetised by intramuscular injection of Zoletil^®^ (Tiletamine 50 mg; Zolacepam 50 mg), and euthanised by captive bolt. Necropsy and sampling were performed following [33].

Handing procedures and sampling frequency were designed to reduce stress and health for subjects, according to European (86/609) and Spanish (R.D. 223/1988, R.D. 1021/2005). No adversal events occurred during the infection and no animals showed signs of illness prior to the study end point.

All experiments were carried out following European, National and Regional Law and Ethics Committee regulation

### Sample processing

Lymph nodes were dissected and stored in sterile containers for further microbiological analyses. Peripheral blood was collected from the conjunctival sinus, cavernous sinus or cava vein of the wild boar using an 18-gauge needle [35]. The collected blood was placed into serum separator tubes and maintained at 4°C in cold boxes until arrival at the laboratory. Serum was obtained by centrifugation at 3.500 rpm for 5 min and conserved at -80°C until further analysis.

### TB severity assessment

To examine the extent of bovine TB-like lesions, wild boar were classified into three groups: i) animals free from TB (TB free), ii) animals with localised gross lesions (mild TB) and iii) animals with disseminated gross lesions (severe TB). Animals showing a mild TB were those with TB lesions in only one location, mainly submandibular or retropharyngeal lymph nodes. Those wild boar with lesions in these lymph nodes or any other organ (e.g., lung, liver, mesenteric lymph nodes and/or spleen) were considered to have severe TB [36].

### Microbiological analysis

For MTC detection, samples of lymph nodes from the head and thorax were pooled, homogenised with sterile distilled water and decontaminated with 0.35% hexadecylpyridinium chloride for 30 minutes [37], centrifuged at 3500 rpm (1068 g) for 30 min and cultured onto Coletsos and 0.2% (w/v) pyruvate-enriched Löwenstein-Jensen media (Biomedics, Madrid, Spain) at 37°C. Isolates were identified by staining for acid alcohol fastness and PCR amplification of the *Mycobacterium* spp. genus-specific 16S rRNA fragment and the MPB70 sequence [38].

### Oxidative stress

To minimise the interpretation bias caused by the use of a single biomarker [25], five different biomarkers were used to assess OS in serum samples of wild boar: SOD, CAT, GPX, GR and TBARS. The latter is considered an indicator of lipid peroxidation whereas SOD, CAT, GPX and GR are endogenous antioxidant enzymes (AE). SOD (U/mg of protein) provides an important antioxidant defence in nearly all cells exposed to ROS generated by cellular immune responses. SOD catalyzes the dismutation of superoxide into oxygen and hydrogen peroxide measured by the inhibition degree of cytochrome C by this enzyme. The method followed for its estimation was that proposed by McCord and Fridovich [39]. CAT (U/mg of protein) catalyzes the decomposition of hydrogen peroxide produced in damaged tissues to water and oxygen and was estimated following the Cohen and Somerson method [40]. GPX (mU/mg of protein) is the general name of an enzyme family with peroxide activity; its biochemical function is to reduce lipid hydroperoxides to their corresponding alcohols and to reduce free hydrogen peroxide to water. Its concentration was determined by estimating NADPH oxidation by the method proposed by Carmagnol et al. [41]. Finally, GR (U/mg of protein) catalyzes the reduction of glutathione disulfide (GSSG) to the sulfhydryl form glutathione (GSH), which is a critical molecule resisting OS and maintaining the redox environment of the cell. This last enzyme was measured following the method described by Cribb et al. [42]. Because ROS have an extremely short half-life, they are difficult to measure directly. Instead, several products of the damage produced by OS can be measured, such as TBARS (nmol MDA/ml), which is formed as a by-product of lipid peroxidation (i.e., as degradation products of fats). An assay of TBARS measures malondialdehyde (MDA), a low-molecular-weight molecule formed by the decomposition of primary and secondary lipid peroxidation products present in the sample. The Buege and Aust method [43] was used to measure MDA. This technique minimises additional oxidation of the sample matrix that would overestimate lipid peroxidation [44]. Biochemical analyses were performed at the Laboratory of Ecophysiology of the *Estación Biológica de Doñana*, Spain (EBD-CSIC) in a microplate, multilabel reader Victor 3 PerkinElmer.

### Statistical analyses

To explore the effects of *M*. *bovis* infection and age on the OS biomarkers in the experimentally infected wild boar (n = 59), the Partial Least Squares Regression approach (PLSR) was used. This statistical tool is an extension of multiple regression analysis and combines features from principal component analysis. PLSR copes better with multicollinearity than generalised linear models [45] and is statistically more robust. The relative contribution of each variable to the derived factors was calculated by means of the square predictor weights. PLSR creates score vectors (also called latent vectors or components) by maximising the covariance between different sets of variables. In the present study, the response variables were the serum concentration of each biomarker of OS (i.e., SOD, CAT, GPX, GR and TBARS) and the explanatory variables were TB status and animal age. The use of this approach minimises the limitations of interpreting a single biomarker [46]. To the authors’ knowledge, this technique has been rarely used in pathological studies; however, a revision of its use in the field of ecology can be found in Carrascal et al. [47]. In a second step, a Kruskal-Wallis post hoc comparison was performed to assess which markers of OS were the most affected by disease severity.

The role of TB, individual, population and environmental sources of OS variation were explored in 61 free-ranging wild boar (24 from NGRPTB, and 37 from CR), using a recursive partitioning approach and regression tree models (RTM). This statistical tool is ideally suited for the analysis of complex ecological data and provides several advantages over other regression techniques, but mainly a better ability to handle missing values in both explanatory and response variables (see De’Ath and Fabricius [48]). In the present case, the response variable was each biomarker of OS whereas the explanatory variables were TB status (free, mild and severe infection), population (NGRPTB or CR), season (using seasonal solstices: autumn, spring, summer or winter), body weight and sex. By these variables, several sources of spatial (e.g., population), temporal (e.g., season) and individual variation (e.g., disease status, body weight and sex) potentially linked to OS biomarker variability were represented. Because boars showing severe TB were more abundant in CR that in NGRPTB, a post hoc tree model exploring the influence of season, sex, body weight and disease status on boars from this population was performed.

For the PLSR analysis the “plspm” package was used [49] and for the RTM analysis the “rpart” statistical package was used [50] (for more information see http://www.gastonsanchez.com and https://cran.r-project.org/web/packages/rpart/rpart). All statistical analyses were performed with the R software version 3. 2. 4 (R Development Core Team, December 2016).

## Results

### Assessment of TB lesions and OS biomarkers in studied wild boar

Globally, 16 out of 61 (26.2%) free-ranging wild boar had TB lesions, with severe TB lesions more frequently found in boars from CR than in NGRPTB. We detected a total of 5 spoligotypes, two in the five animals of NGRPTB (SB0294 (n = 4) and SB0415 (n = 1) and three in the 11 of CR (SB0119 (n = 1), SB0339 (n = 2) and SB0295 (n = 8)). A part of *M*. *bovis*, *M*. *caprae* was detected in one animal of NGRPTB, in CR all *Mycobacteria* were *M*. *bovis*. For more information see [51] regarding NGRPTB animals and [52] regarding CR wild boar. TB lesions were generated in all experimentally infected wild boar. All cases of suspected TB by gross examination were confirmed by means of bacteriological analyses. No *M*. *bovis* was detected in animals with a lack of gross lesions compatible with TB.

Table 1 summarises age, sex and TB lesion extent in experimental and free-ranging wild boar. In experimentally infected animals, 16 juveniles and four yearlings were infected with severe TB, and 14 juveniles and 10 yearlings were infected with mild TB; control animals (two juveniles and 13 yearlings) were negative for *M*. *bovis*. In free-ranging wild boar from CR, two adults and two yearlings had severe TB, two yearlings and five juveniles displayed mild TB lesions, and 13 juveniles and 13 yearlings were TB free. No wild boars from NGRPTB showed severe TB status, while one piglet, two yearlings and two adults had mild TB and one piglet, three juveniles, four yearlings and 11 adults were free from MTC infection.

**Table 1:** Summary of the epidemiological values of the wild boar experimentally and naturally infected with *Mycobacterium tuberculosis* complex. Data from NI animals came from two populations, Ciudad Real (CR, n = 37)) and The Natural Game Reserve Ports de Tortosa i Beseit (NGRPTB, n = 24).

		Age				Sex		TB status		
		Piglet	Juvenile	Yearling	Adult	Male	Female	TB free	Mild TB	Severe TB
Experimentally infected	(n = 59)	0	32	27	0	42	17	15*	20**	24**
Free-ranging	CR	0	18	17	2	22	15	26	7	4
	NGRPTB	2	3	6	13	13	11	19	5	0

*Control wild boar;

**M. *bovis* inoculated wild boar

Tables 2 and 3 summarise descriptive values for OS biomarkers of experimental and free-ranging wild boars. Experimental TB free animals had higher values of AE; in contrast, the highest differences for free-ranging boars were detected between populations (CR versus NGRPTB) and not between TB groups.

**Table 2:** Descriptive statistics for oxidative stress biomarkers. Superoxide dismutase (SOD), catalase (CAT), glutathione peroxidase (GPX), glutathione reductase (GR) and thiobarbituric acid reactive substances (TBARS), in serum from wild boar experimentally infected with *M*. *bovis*. The SOD, CAT, GPX and GR enzymes were measured in units of activity per mg of protein (U/mg), and TBARS in nanomoles of malondialdehyde per ml (nmol MDA/ml).

		Mean	SE	Min—Max
SOD (U/mg)	TB free	2.92	0.32	0.97–5.82
	Mild TB	1.75	0.44	0.08–12.04
	Severe TB	1.34	0.23	0.30–4.50
CAT (U/mg)	TB free	43.15	14.44	5.45–180.00
	Mild TB	32.23	8.47	7.54–202.19
	Severe TB	13.78	1.19	5.38–27.81
GPX (mU/mg)	TB free	17.83	1.29	9.36–29.31
	Mild TB	14.3	2.26	2.82–46.26
	Severe TB	12.04	1.94	2.71–48.74
GR (U/mg)	TB free	0.11	0.02	0.039–0.393
	Mild TB	0.13	0.02	0.036–0.387
	Severe TB	0.12	0.01	0.052–0.286
TBARS (nmol MDA/ml)	TB free	10.25	1.16	5.11–22.88
	Mild TB	8.49	0.68	3.57–20.84
	Severe TB	8.9	1.02	3.60–23.23

SE: standard error; TB free: TB negative animals; Mild TB: animals with localised lesions in lymph nodes; Severe TB: animals showing generalised lesions in lung, liver, mesenteric lymph nodes and/or spleen.

**Table 3:** Descriptive statistics for superoxide dismutase (SOD), catalase (CAT), glutathione peroxidase (GPX), glutathione reductase (GR) and thiobarbituric acid reactive substances (TBARS), in serum from wild boar naturally affected by TB.

		Population	Mean	SE	Min—Max
SOD (U/mg)	TB free	NGRPTB	4.04	0.40	1.69–7.33
		CR	3.01	0.44	0.16–8.96
	TB	NGRPTB	2.64	0.41	1.26–3.52
		CR	2.54	0.48	0.99–5.83
CAT (U/mg)	TB free	NGRPTB	54.66	3.25	38.04–89.04
		CR	20.90	3.5	5.72–78.16
	TB	NGRPTB	46.87	2.62	40.50–54.77
		CR	11.71	2.08	3.34–22.21
GPX (mU/mg)	TB free	NGRPTB	10.5	1.2	3.6–23.2
		CR	15.63	1.16	4.17–30.42
	TB	NGRPTB	9.87	2.1	4.85–14.89
		CR	13.5	1.2	7–18.6
GR (U/mg)	TB free	NGRPTB	0.262	0.035	0.098–0.567
		CR	0.0950	0.0078	0.0504–0.2014
	TB	NGRPTB	0.1924	0.0395	0.113–0.34
		CR	0.0857	0.0097	0.0448–0.1465
TBARS (nmol MDA/ml)	TB free	NGRPTB	4.99	0.46	2.82–12.31
		CR	13.30	0.68	9.21–20.23
	TB	NGRPTB	3.54	0.32	2.96–4.62
		CR	11.63	1.08	5.71–15.53

The SOD, CAT, GPX and GR enzymes were measured in units of activity per mg of protein (U/mg), and TBARS in nanomoles of malondialdehyde per ml (nmol MDA/ml). SE: standard error; TB free: TB negative animals; TB: animals with localised lesions in lymph nodes or generalised lesions in lung, liver, mesenteric lymph nodes and/or spleen. CR: Ciudad Real; NGRPTB: National Game Reserve Ports Tortosa i Beceit.

### Oxidant/antioxidant status in experimentally M. bovis-infected wild boar

**Fig 1:**
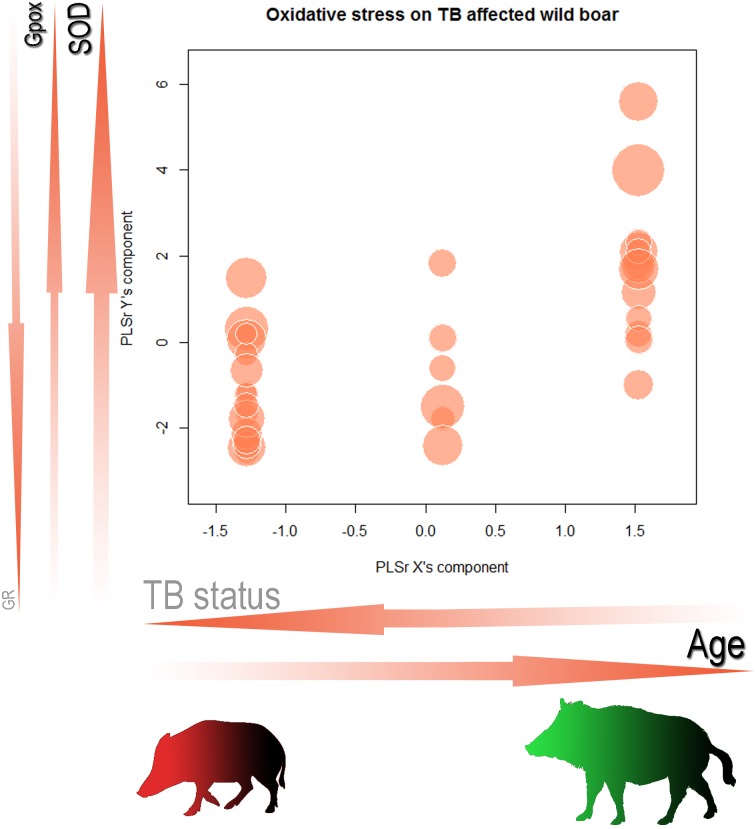
Bubble plot representing the relationships between a PLSR X component describing age and tuberculosis status and the PLSR Y component describing antioxidant capacity of 59 wild boar (*Sus scrofa*) experimentally infected with *M*. *bovis*. Font and arrow size indicate the weight of each variable whereas font colour indicates either the increase (black) or the decrease (grey) of the score components. Bubble diameter represents TBARS concentration (nmol MDA/ml), an oxidation biomarker. Thus, for proper interpretation both the bubble size and its position should be taken into account. Large bubbles at low PLSR Y scores suggest boars with oxidative damage whereas the same bubbles at high PLSR Y scores indicate that individuals are able to compensate for the increase in oxidation (high antioxidant /oxidant value). Each bubble represents individuals having the same X and Y score values. The red wild boar shape at the bottom represents a young wild boar suffering from oxidation due to *M*. *bovis*, whereas the green shape represents a TB-free adult wild boar in good antioxidant status.

Since the initial PLSR model consisted of a PLSR Y’s component including all of the OS biomarkers, only GPX, SOD and GR were retained in the final model. This model showed that 17.6% of the observed variability of wild boar antioxidant capacity was significantly (Q^2^ = 0.132, p-value < 0.05) correlated with the PLSR X’s component represented by both disease status and the age of animals (Fig 1). Both the age and TB status contributed similarly to the PLSR X’s component (Table 4). In the Y’s component, however, SOD, GPX and GR contributed in decreasing order of importance (Table 4 and Fig 1). In general, all the antioxidant biomarkers were negatively related to the TB status indicating low serum concentrations of these enzymes in individuals with severe TB (Table 2).

**Table 4:** Summary of the partial least squares regression between oxidative stress and tuberculosis status and age.

PLSR component	Predictors	Loads	Weights	VIP	Corr. Xu	Corr. Xt
X	Age	0.71	0.71	1.01	0.6	0.91
	TB status	-0.71	-0.71	0.99	-0.6	-0.91
Y					**Corr. Yt**	**Corr. Yu**
	GPX	0.21	—	—	0.27	0.65
	SOD	0.49	—	—	0.63	0.92
	GR	-0.18	—	—	-0.24	0.2

The PLSR Y component representing antioxidant status by the enzymes superoxide dismutase (SOD), glutathione reductase (GR) and glutathione peroxidase (GPX) and the PLSR X component represented by the age and the tuberculosis status (i.e., TB free, Mild TB and Severe TB) of experimental wild boars. VIP: variable importance in projection; Corr. Xu: correlation between each explanatory variable in the X’s component and the Y’s component; Corr. Yt: correlation between each response variable in the Y’s component and the X’s component; Corr. Xt: correlation between explanatory variables and X’s component; Corr. Yu: correlation between response variables and the Y’s component.

**Fig 2:**
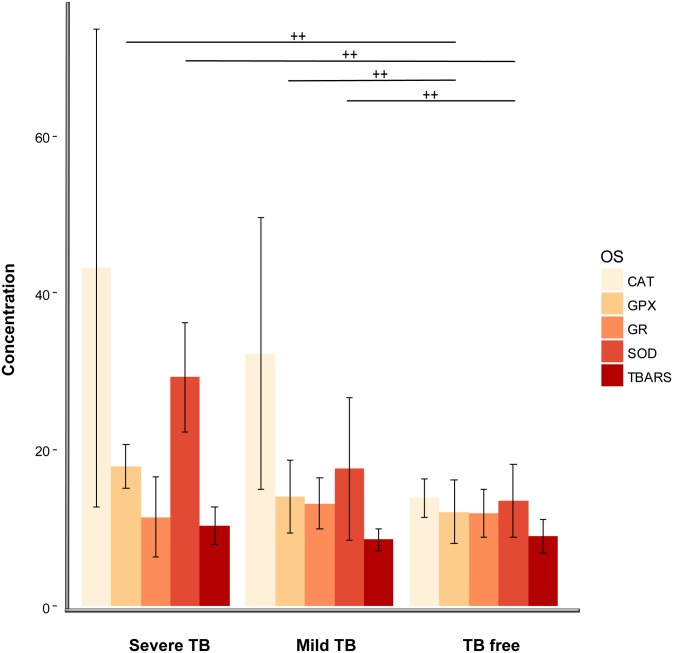
Mean concentration and associated standard error (SE) of lipid peroxidation (TBARS) and endogenous antioxidant enzymes (CAT, SOD, GPX and GR), in serum from wild boar experimentally infected with *M*. *bovis*. Concentrations of SOD, CAT and GR enzymes were measured in units of activity per mg of protein (U/mg), GPX in mU/mg, and TBARS in nanomoles of malondialdehyde per ml (nmol MDA/ml). Results of both SOD and GR were multiplied by ten for graphic representation. Wild boar were divided into three groups: TB free n = 15, Mild TB (localised lesions in lymph nodes) n = 20 and Severe TB (generalised lesions in lung, liver, mesenteric lymph nodes and/or spleen) n = 24. Wiskers represent 95% confidence intervals and the horizontal lines the results of a post hoc Kruskall wallis test. Statistically significant differences, at α = 0.05 are indicated by crosses.

SOD concentrations were the most affected by TB (chi-squared = 17.58, df = 2, p-value = 0.0001), with TB-free animals between 1.6 to 2.19 times higher than in boars showing mild or severe TB (Table 3 and Fig 2). To a lesser degree, GPX concentrations also varied according to TB status (chi-squared = 7.16, df = 2, p-value = 0.028), being 1.25 to 1.48 times higher in TB-free boars compared to mild or severely TB-affected individuals. Despite the fact that CAT followed the same pattern, the post hoc Kruskall-Wallis test was not significant (chi-squared = 1.85, df = 2, p-value = 0.3969), probably because of the large standard error (SE = 14.44) and the wide rank of CAT values both in TB-free and affected animals (Table 3). Along the same lines, no differences in GR concentrations were observed between groups (chi-square = 3.09, df = 2, p-value = 0.213).

### Oxidant/antioxidant status in free-ranging wild boar

**Fig 3:**
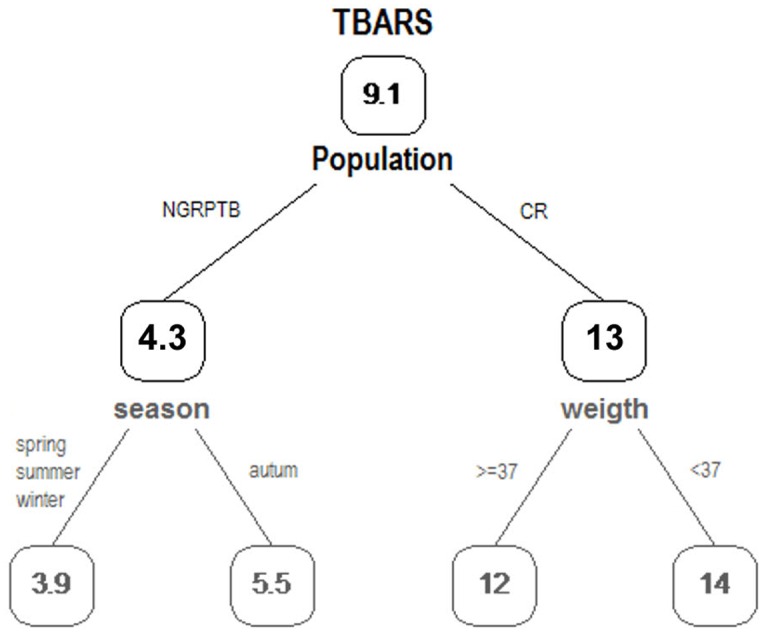
Regression tree model of lipid peroxidation (TBARS). The regression tree model used the parameter anova. The chosen partition was that which minimizes the sum of squared residuals. In this case the complexity parameter (CP) was 0.0001 (when de gain of the coefficient of determination was less than CP no additional partitions will be formed). The pruned tree was included (with only one node and with the lower cross-validation error) in black and the complete tree with the best response variable in gray. The values inside the boxes were TBARS serum values of wild boars (nmol MDA/ml). The explicative variable “population” divides the TBARS serum values with a mean of 12.82 nmol MDA/ml for CR animals and 4.29 for NGRPTB wild boars.

The best RTM of the role of environmental and individual drivers of OS in both healthy and TB-affected wild boars were built with population as a single split factor (Table 5). In fact, mean concentrations of the five OS biomarkers differed mainly between populations, with the other factors (i.e., season and body weight) being irrelevant. As an example, for TBARS, which in turn built the model with the best fit (lower relative error and higher R^2^, Table 5 and Fig 3), the mean concentration in boars from CR was 2.7 times higher than in NGRPTB animals. Along the same lines, mean GR concentration in boars from CR was 14.97 and 10.38 mU/mg of protein for animals from NGRPTB. For CAT, mean concentration in CR boars was 24.31 and 53.04 U/mg of protein in NGRPTB animals. Finally, although SOD variability was explained by the effects of the season, the high relative error and the low fit did not allow for model interpretation.

**Table 5:** Summary of five regression tree models for evaluate the importance of individual and environmental sources of oxidative stress variation.

Response variable	Population	Cross-validation error	Relative error	Complexity parameter	R^2^	VI
TBARS	NGRPTB + CR	0.30	0.28	0.02	0.72	P(46) > S(34) > BW(19) > TB(1)
	CR	1.06	1	0.11	0	BW(48) > A(40) > TBL(12)
SOD	NGRPTB + CR	0.96	0.86	0.05	0.14	S(68) > BW(24) > P(3) > TB(3) > G(2)
	CR	1.03	1	0.08	0	BW(47) > A(44) > TBL(8)
CAT	NGRPTB + CR	0.86	0.77	0.02	0.23	P(41) > S(29) > BW(27) > TB(3)
	CR	1.02	0.92	0.001	0.08	BW(47) > A(44) > TBL(8)
GPX	NGRPTB + CR	0.88	0.84	0.07	0.16	P(37) > BW(30) > S(26) > TB(4) > G(3)
	CR	0.99	0.73	0.001	0.27	BW(62) > A(25) > TBL(6) > G(5) > S(1)
GR	NGRPTB + CR	0.54	0.44	0.01	0.56	P(43) > S(32) > BW(20) > TB(3) > G(1)
	CR	1.13	1	0.14	0	BW(87) > TBL(9) > S(5)

We explore the relationships between population (P), season (S), body weight (BW), tuberculosis status (TB), gender (G) and age (A) and biomarkers of oxidative stress: thiobarbituric acid reactive species (TBARS), superoxide dismutase (SOD), catalase (CAT), glutathione peroxidase (GPX) and glutathione reductase (GR) serum enzymes of wild boars naturally infected with *M*. *bovis*. Animals come from two populations: the National Game Reserve of Ports de Tortosa i Beseit (NGRPTB), and from Ciudad Real (CR). In CR, severe TB is more frequent than in NGRPTB, hence we performed a post hoc tree model using only boars from this population (shadow lines). The R^2^ is the proportion of observed variability explained by a given tree model. Cross-validation error: medium error of 10 cross validations; Relative error: is the part of the variance not explained by the tree model; Complexity parameter: minimum complexity benefit that must be gained at each step in order to make a split worthwhile. The default is 0.001; Variable importance (VI): sum of the goodness of fit measures for each split for which it was the primary variable, plus goodness * (adjusted agreement) for all splits in which it was a surrogate variable.

The post hoc regression tree analysis performed in CR, where severe TB was common among boars, showed that body weight was more important than disease severity for explaining the observed variability of OS biomarkers. The model fit, however, was so poor that conclusions could not be drawn (Table 5).

## Discussion

Based on our results, a positive oxidant/antioxidant balance (i.e., low antioxidant enzyme production for a given concentration of oxidant substances) in *M*. *bovis*-infected boars can be detected under experimental conditions but not in free-ranging animals exposed to environmental variation. The results obtained in our experimental challenge are in line with the basic pathophysiological mechanism proposed in other vertebrate models. In mice and humans, for example, an increase in OS is the main mechanism limiting *Mycobacteria* spp. multiplication, thus preventing the appearance of TB-like gross lesions [52, 53]. At the same time, the bacterium limits the exposure to free radicals by increasing the expression of antioxidant enzymes [54, 55], which slows down macrophage ROS and RNS production [9, 8]. These “attack and counterattack” interactions are key drivers for maintaining the TB granuloma in a latent form that can be reversed in case of immune depression [30].

Our challenged boars may have experienced the same pathophysiological process since individuals with TB gross lesions showed low SOD and GPX concentrations for a given degree of oxidation (TBARS). In agreement with observations made in other vertebrate models [4], in which AE mobilisation was needed to maintain OS homeostasis, our wild boars may show high SOD and GPX depletion to protect tissues from the intermediate production of macrophage reactive nitrogen during the severe form of the disease [9]. Since oxidative damage depends on whether AE production is sufficient to compensate for oxidative damage [22], we can assume that our challenged wild boars developing TB-like gross lesions are suffering from oxidative stress.

This antioxidant capacity depression has also been observed in TB [56] confirming the value of OS biomarkers as indirect indicators of bacteria proliferation during TB infection. Though it was ultimately not retained in our PLSR model, CAT concentration was also lower in individuals showing TB gross lesions. Previous research has also observed few variations in CAT concentrations between TB-affected and healthy patients [57].

Age was also linked to changes in the oxidant/antioxidant balance in our experimental animals. In the last few decades, several works have shown that aging *per se* is a consequence of oxidative damage [23, 58]. In our case, the PLSR model exemplifies that SOD and GPX were positively but poorly correlated with age. In contrast, GR was negatively and highly correlated with age, suggesting that yearling boar had lower levels of GR in serum than juveniles. In wild boar, glutathione levels were not sensitive to senescence and TBARS had a curvilinear pattern in relation to age [19]. Similar inconclusive results have been obtained in domestic pigs infected by porcine reproductive and respiratory syndrome virus [59]. In this study, the age group (weaners, fatteners or finishers) influenced concentration of AE in infected animals. The relationship between OS biomarkers and the aging process in wild boar, as well as pigs, is still unclear.

On the other hand, CR free-ranging wild boar had lower AE and higher TBARS levels compared to the NGRPTB boar. This would imply a higher oxidative imbalance and damage because the mobilisation of AE was lower than their utilization and maybe lower than their need [22]. However, TB status did not appear to be an important factor in explaining the observed variability in OS biomarkers; the fact that TB prevalence was lower (16 positive out of 61 wild boar) and mostly with localised lesions (12), could be an explanation. In fact, no wild boar from NGRPTB and four from CR had severe TB lesions. The main variable to explain TBARS, GPX, CAT and GR variability was population (CR or NGRPTB) and not TB status. For SOD, however, the main explanatory variable was the season. Intrinsic (genetic [60], life-history traits [61] or different TB granuloma phases [30]) as well as extrinsic factors (such as other pathogens [62], population density, environmental contaminants [63], season [64] and diet [65], among others) may hide TB effects [22]. Since all of these factors are related, it is difficult to recognize individual effects on OS. The fact that TB status did not appear to be a significant factor in the *ad hoc* model of CR animals, where extrinsic factors were reduced, suggests that intrinsic factors may also play an important role in OS levels.

## Conclusions

Although TB causes the oxidant/antioxidant imbalance in experimental conditions, oxidative stress goes unnoticed in free-ranging *M*. *bovis*-infected animals. Accordingly, other environmental factors causing OS, such as parasites and/or food availability, should have a greater impact on OS than TB itself. Despite the growing interest in biomarkers of oxidative status for assessing the health status of individuals in conservation programmes, the regular use of these biomarkers to assess the impact of wildlife diseases is in its infancy. Biomarkers of oxidative status can help to quantify fitness costs of diseased animals but much work remains to be done to understand the natural sources of variation affecting these promising physiological indicators.

## Supporting Information

S1 File Data on experimentally infected wild boar. (XLSX)

S2 File Data on free-ranging wild boar. (XLSX)
