# Correction: Oxidative Stress in Wild Boars Naturally and Experimentally Infected with *Mycobacterium bovis*

**DOI:** 10.1371/journal.pone.0174075

**Published:** 2017-03-09

**Authors:** Diana Gassó, Joaquín Vicente, Gregorio Mentaberre, Ramón Soriguer, Rocío Jiménez Rodríguez, Nora Navarro-González, Asta Tvarijonaviciute, Santiago Lavín, Pedro Fernández-Llario, Joaquim Segalés, Emmanuel Serrano

Fig 2 is incorrect. The axis labels Severe TB and TB free are inappropriately switched. The proper label order should read: TB free, Mild TB, Severe TB. The authors have provided a corrected version of Fig 2 here.

**Fig 2:**
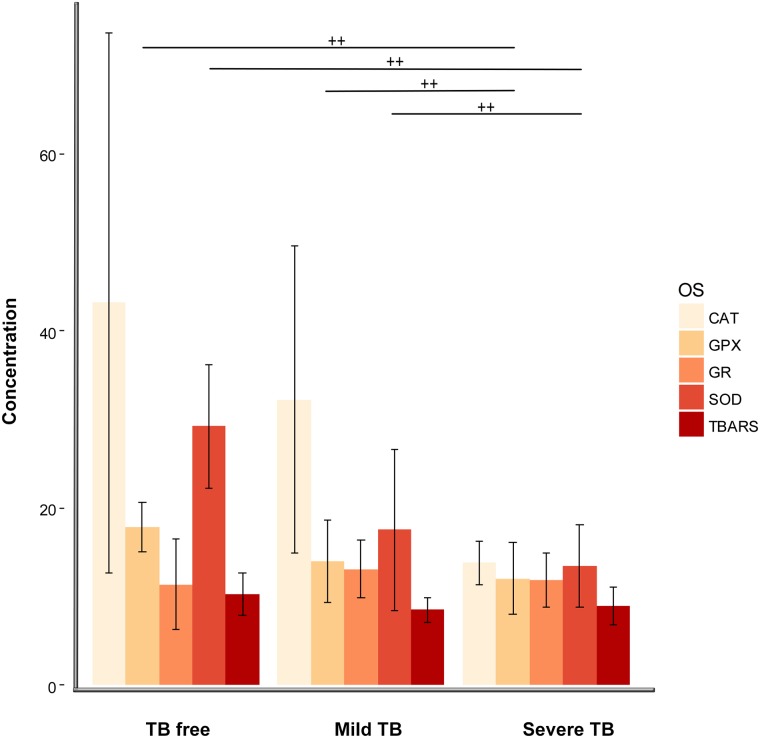
Mean concentration and associated standard error (SE) of lipid peroxidation (TBARS) and endogenous antioxidant enzymes (CAT, SOD, GPX and GR), in serum from wild boar experimentally infected with *M*. *bovis*. Concentrations of SOD, CAT and GR enzymes were measured in units of activity per mg of protein (U/mg), GPX in mU/mg, and TBARS in nanomoles of malondialdehyde per ml (nmol MDA/ml). Results of both SOD and GR were multiplied by ten for graphic representation. Wild boar were divided into three groups: TB free n = 15, Mild TB (localised lesions in lymph nodes) n = 20 and Severe TB (generalised lesions in lung, liver, mesenteric lymph nodes and/or spleen) n = 24. Wiskers represent 95% confidence intervals and the horizontal lines the results of a post hoc Kruskall wallis test. Statistically significant differences, at α = 0.05 are indicated by crosses.
